# Value of spiral CT multi-parameter combined preoperative evaluation of microvascular invasion in small liver cancer

**DOI:** 10.12669/pjms.37.6-WIT.4851

**Published:** 2021

**Authors:** Kun Li, Yongjun Peng, Hongzhe Tian, Hailin He

**Affiliations:** 1 Kun Li, Associate Chief Physician. Department of Medical Imaging, Baoji Center Hospital, Baoji 721008, Shaanxi, China; 2 Yongjun Peng, Chief Physician. Department of Radiology, Zhuhai People’s Hospital, Zhuhai 519000, Guangdong, China; 3 Hongzhe Tian, Associate Chief Physician, Master of Medicine, Department of Medical Imaging, Baoji Center Hospital, Baoji 721008, Shaanxi, China; 4 Hailin He, Associate Chief Physician. Department of Medical Imaging, Baoji Center Hospital, Baoji 721008, Shaanxi, China

**Keywords:** Multi-slice spiral CT, Preoperative, Microvascular invasion, Small liver cancer, Hepatocellular carcinoma

## Abstract

**Objective:**

To explore the value of multi-slice spiral CT (MSCT) in predicting microvascular invasion in hepatocellular carcinoma (HCC).

**Methods:**

The CT and clinical data of 102 patients with HCC were collected for retrospective analysis from January 2018 to December 2020 at Baoji Center Hospital, China. They were divided into two groups based on the pathological results with or without microvascular invasion. The independent sample t-test was used to compare the age, alpha-fetoprotein (AFP) value, tumor size, and tumor enhancement of the two groups. CT value; χ^2^ test was used to compare gender, hepatitis type, liver function classification, degree of classification, degree of tumor smoothness, envelope, peripheral enhancement, etc. between the two groups.

**Results:**

There were 52 cases of non-microvascular invasion and 50 cases of microvascular invasion. The tumor size, grade, degree of margin, capsule, portal vein CT value, and peripheral enhancement were related to microvascular invasion.

**Conclusion:**

Microvascular invasion of HCC can be predicted by MSCT manifestations before surgery.

## INTRODUCTION

Many researchers have tried to evaluate microvascular invasion using methods such as hepatic angiography and enhanced MRI. However, the imaging manifestations of microvascular invasion of HCC are not clear. This study aims to predict the microvascular invasion of HCC by multi-slice spiral CT (MSCT) imaging.^1^-^3^ At this stage, lung CT imaging is still the primary method for diagnosing lung cancer in China. However, when tuberculosis and lung cancer coexist, lung cancer diagnosis and treatment are easily delayed due to similar symptoms. Therefore, the lung CT imaging of the two is identified. The characteristics are of great significance for guiding the clinical diagnosis of tuberculosis with lung cancer.

## METHODS

Retrospective analysis of the image data of all patients who underwent radical resection of HCC in the Baoji Center Hospital, from January 2018 to December 2020 was performed after taking IRB approval on March 24, 2021.

### Inclusion criteria

(1) no macrovascular tumor thrombosis was found in all image data There is only one isolated mass; (2) The interval between CT examination and surgery should be less than 1 month. Finally, 102 patients with HCC were selected as the research subjects, 76 males and 26 females, aged 37 to 85 years, with an average age of 61.8 years, 55 patients with hepatitis B, 36 patients with hepatitis C, four patients with hepatitis B and C, seven cases of alcoholic hepatitis. Child-Pugh classification of liver function: 98 cases of grade A, three cases of grade B, one case of grade C. 4,5

### CT examination

The TOSHIBA Aquilion 64-slice spiral CT scan was used. The scanning layer thickness was 5mm, the pitch was 0.75, the tube voltage was 120kV, and the tube current was 180-220mAs. The whole liver was scanned first and then the scan was enhanced. It was 1.5ml / kg body weight. It was injected through a superficial vein of the elbow with a double-barreled high-pressure syringe at a flow rate of about 3ml / s. The arterial phase, portal vein phase, and delayed phase scan were performed at 30s, 60s, and 120s, respectively.

### Imaging Analysis

Two deputy chief physicians observed CT images on the workstations and gave a diagnosis. The tumor size, margins, capsules, enhancement methods, and peripheral enhancement were evaluated. When inconsistencies were negotiated with each other. Smooth tumor edges appear as smooth arcs on imaging images in all directions. The envelope is a ring-shaped high-density shadow with a clear edge of the tumor at the portal vein or delayed stage. The envelope surrounding the tumor ≥40% is recorded as an envelope; <40% is recorded as an envelope-free. When measuring CT values, avoid areas such as hemorrhage, necrosis, and cystic changes. Take three measurements and take the average value. Select the same level for each phase of the same lesion. The halo sign needs to be observed around the tumor enhancement. The halo sign is a ring-shaped or band-like enhancement of the liver parenchyma around the tumor at the late stage of arteries or early portal veins, and the delay period decreases with equal density.^6^

### Pathological analysis

The pathologist observed all the postoperative pathological sections and found that tumor thrombus aggregated in the small blood vessels (central vein, portal vein branch and capsular venule), or cancer cells appeared in the vascular endothelial layer and smooth muscle layer, then it was determined as HCC. Microvascular invasion. According to Edmondson Steiner classification (grade 1 to 5), the degree of tumor differentiation was determined.

### Statistical analysis

SPSS 18. 0 statistical software was used to analyze. Micro-vascular invasion was divided into two groups according to pathological results. Independent sample t test was used to compare the age, alpha-fetoprotein (AFP) value, tumor size, and tumor-enhanced CT value of the two groups of patients. χ^2^ test was used to compare the sex, hepatitis type, liver function grade, tumor grade, tumor smoothness, envelope, and halo sign between the two groups. The difference was statistically significant with P <0.05.^7^,^8^

## RESULTS

**Fig.1:**
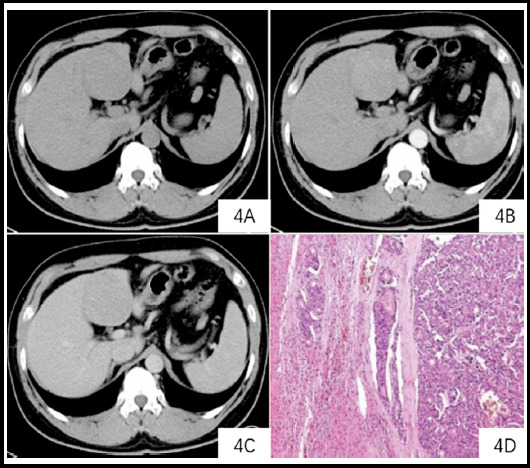
CT scan of the left lobe of the liver shows a circular low-density lesion. ***Note:*** A 51-year-old man with a CT scan of the left lobe of the liver showed a circular low-density lesion of approximately 5.8 cm x 5.4 cm, with a CT value of approximately 45 HU (4A); uneven enhancement of arterial lesions, and a CT value of 69 HU (4B); The CT value of portal vein (4C) was 81HU. Pathology (HE staining x 200) showing enveloped vascular cancer thrombus (4D).

**Fig.2:**
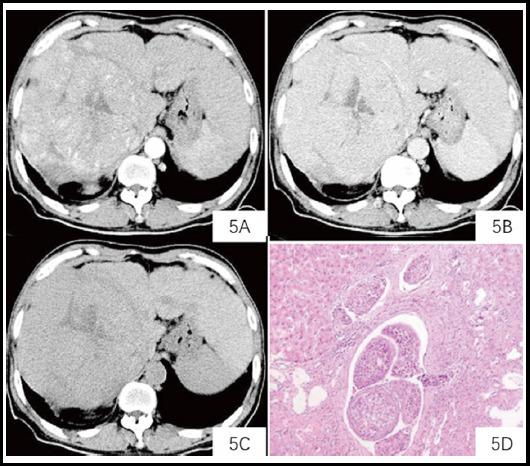
A plain scan of the right lobe of the liver shows a low-density mass. ***Note:*** The patient was male, 58 years old. A plain scan of the right lobe of the liver revealed a low-density mass of 10.1 cm x 11.5 cm. The CT value was approximately 49 HU (5A). The solid components in the arterial phase were significantly enhanced, and the CH value was approximately 91 HU (5 B). The CT value in the portal vein phase (5C) was 78 HU. Pathology (HE staining × 200) showed tumor thrombus in the small vein (5E).

According to the pathological results, they were divided into a non-microvascular invasion group (52 cases) and a microvascular invasion group (50 cases). The clinical and imaging data of the two groups of patients are compared in Table-I and Fig. 1 and 2. There was no significant difference between the two groups in terms of age, gender, hepatitis type, liver function classification, AFP value, arterial phase and delayed phase CT value (P> 0.05); in tumor pathological classification, size, margin, envelope. There was a statistically significant difference in CT value and peripheral enhancement in portal vein stage (P <0.05).

**Table-I:** Relationship between clinical and imaging data and microvascular invasion.

*Clinical and imaging data*	*Non-microvascular invasion group*	*Microvascular invasion group*	*t-value or χ^2^ value*	*P-value*
Age	63.31±5.02	65.96±5.80	2.473	0.362
Male (example)	37	39	0.629	0.428
Hepatitis B (example)	28	27	0.000	0.988
Hepatitis C (example)	19	17	0.072	0.789
Hepatitis B + C (Example)	2	2	−	1
Alcoholic hepatitis (example)	3	4	−	0.713
*Liver function classification*				
A (example)	51	47	−	0.358
B (example)	1	2	−	0.614
AFP (μg/mL)	416.29±576.31	337.84±529.07	0.715	0.476
*Edmondson Steiner grading*				
1/2 level (example)	7/25	2/18	4.732	0.030
Level 3/4 (example)	14/6	20/10	−	−
Tumor size (cm)	3.67±0.83	4.40±1.32	3.218	0.002
Tumor edges are not smooth (example)	19	35	11.456	0.001
Tumor has an envelope (example)	39	26	5.833	0.016
Arterial CT value (HU)	79.88±11.78	75.94±9.39	1.866	0.065
CT value of portal vein (HU)	70.62±10.96	84.80±12.50	6.102	<0.001
Delay CT value (HU)	69.17±12.70	72.04±9.50	1.29	0.201
Halo sign (example)	29	38	4.629	0.031

## DISCUSSION

The results of this study show that the CT value of arterial tumors in the non-microvascular invasion group is slightly higher than that in the microvascular invasion group; the CT value of portal vein tumors in the non-microvascular invasion group is significantly lower than that in the microvascular invasion group. It showed that the portal vein phase of the tumor in the microvascular invasion group continued to strengthen, and the CT value continued to increase. The enhancement method was the continuous enhanced type in the portal vein phase. The CT value was significantly higher than that in the non-microvascular invasion group. The blood supply of the tumor was closely related to the enhancement method.

The enhancement method suggests that the tumor is dually supplied by the hepatic artery and the portal vein. After microvascular invasion, the tumor will accelerate the release of tumor angiogenesis promoting factors, such as hypoxia-inducible factor-1α (HIF-1α) and vascular endothelial growth factor (VEGF). New blood vessels will diversify the blood supply to the tumor. The tumor enhancement method reflects the characteristics of blood supply. When the hepatic artery and portal vein are dual-supply, the arterial phase and the portal vein phase are gradually strengthened, and the strengthening duration is longer. 9,10 Microvascular invasion tumors continuously release a large amount of HIF-1α and VEGF, and induce many micro angiogenesis. The enhancement of the arterial phase and the portal vein phase is mainly affected by angiogenesis. The delayed phase enhancement is mainly related to the extravascular space and the permeability of blood vessels. These factors are related to HIF-1α and VEGF, which lay the pathophysiology of tumor enhancement. Therefore, HCC microvascular invasion can be predicted by the enhancement method of tumor portal vein phase and CT value increase. The non-smooth tumor edge has implications for predicting microvascular invasion. 11,12 It is believed that solitary masses with nodular protrusions and multiple nodular fusion are more likely to be accompanied by microvascular invasion than a single mass. However, preoperative CT imaging is difficult to distinguish between multiple nodules and solitary masses with nodule protrusions. 13,14 The results of this study showed that intra-tumoral arteries were statistically significant in predicting microvascular invasion. The intra-tumoral artery sign was more likely to appear in the microvascular invasion group than in the non-microvascular invasion group. It was in line with the study of Segal 15, which proved that this sign was related to angiogenesis and cell proliferation. The peri-tumor enhancement, low-density halo sign, and tumor-to-liver difference were found to be not statistically significant in predicting HCC microvascular invasion. Some studies have used enhancement around the tumor, low-density halo sign, and tumor-liver difference as parameters, suggesting an increased risk of tumor microvascular invasion. This may be due to the different imaging methods and the low percentage of surrounding tumor enhancement on dynamic CT images. The results still need to be confirmed using an expanded sample size. At the same time, the presence of tumor envelope and microvascular invasion did not show a significant correlation. However, studies have found that, the fibrous envelope in HCC is a favorable prognostic factor because the envelope can prevent liver cancer from invading the adjacent liver parenchyma. However, Adachi 16 showed that, fibrous enveloped blood vessels were often invaded by cancer cells, and pointed out that the presence of fibrous envelope was a predictor of portal invasion. The relationship between the existence of tumor capsule and the occurrence of MVI still remains academically controversial, and further research is needed. 17,18

## CONCLUSIONS

In short, the size of HCC tumors, Edmondson Steiner classification, marginal, envelope, portal vein enhancement characteristics, peripheral enhancement are closely related to microvascular invasion. When the tumor is larger, Edmondson Steiner classification is higher, matte edges, no envelope, portal vein Continued intensification, enhanced CT values and peripheral intensification are often suggestive of microvascular invasion when there are halo signs. Patients need more active treatment, expanding the scope of surgery or combining with adjuvant treatment to reduce the recurrence rate.

### Author`s Contribution

**KL** conceived the study, literature review, data analysis, drafting the manuscript.

**YP and HT** helped in design, data collection, article drafting & critical revision.

**HH** takes the responsibility and is accountable for all aspects of the work in ensuring that questions related to the accuracy or integrity of any part of the work are appropriately investigated and resolved.
